# Impact of Social Connections on Well-Being of Older Adults: Relationships, Communities, and Systems of Support

**DOI:** 10.3390/healthcare14183048

**Published:** 2026-09-17

**Authors:** Kateryna Metersky, Marco Nanfara, Lixia Yang

**Affiliations:** 1 Daphne Cockwell School of Nursing, Toronto Metropolitan University, Toronto, ON M5B 2K3, Canada; 2 Department of Psychology, York University, Toronto, ON M3J 1P3, Canada; 3 Department of Psychology, Toronto Metropolitan University, Toronto, ON M5B 2K3, Canada

## 1. Introduction

Social connection is a crucial component of health and well-being for any individual, especially older adults, who are often at increased risk for social isolation [Contribution 1]. Relationships with family, friends, caregivers, and communities can provide emotional support and a sense of belonging [Contribution 2]. However, the presence of others does not always ensure meaningful connection. The value of support depends on the type of support provided and the quality of the relationship. The COVID-19 pandemic disrupted opportunities for social interaction and engagement and thus highlighted the importance of community participation in times of change. The effects on social connection continued beyond periods of physical distancing, even in the present. In this context, it is very important to understand how social connections can be rebuilt and maintained in later life [Contribution 3]. This is especially significant for migrant older adults who may experience social connection in very different ways. The process of migration, language barriers, and restricted access to culturally appropriate services can shape the availability and meaning of social support in this population [Contributions 4,5]. Meaningful connections therefore depend on relationships that provide trust, recognition, and opportunities to participate, rather than relying only on the frequency of social contact.

This Special Issue brings together ten contributions (Table S1) examining social connection and well-being among diverse older populations and across various cultural, geographic, and caregiving contexts. The collection includes review and research articles using quantitative, qualitative, and mixed-methods designs. The articles examine social support, caregiving, and access to responsive services. Together, they demonstrate how late-life well-being is shaped by relationship quality and features, community participation, and broader systems of support. This editorial considers how these contributions extend current understandings of social connection among older adults, and highlights shared implications for research, practice, and service.

## 2. Current State of Knowledge

Current research conceptualizes social connection as a multifaceted component of well-being in later life [Contributions 1–5]. It also considers the size and composition of social networks rather than only the presence or frequency of social contact. The extent of connection may be reflected in network size and opportunities for participation, while its value depends on whether relationships provide trust, practical support, and a sense of belonging. Recent research describes the dimensions of social connection as structural, functional, and qualitative. Each connection may have different implications for health and well-being [1]. These aspects are related but not interchangeable, as people may feel unseen and lonely despite regularly interacting with others [Contribution 5]. The source of support and conditions in which relationships develop affect the influence of social connections [Contributions 2,3]. The Convoy Model of Social Relations supports this perspective by suggesting that people move through life with changing networks of family and non-family relationships. The composition and functions of these relationships may shape their health and well-being [2]. The benefits of social support may vary depending on personal resilience and the role a relationship serves [Contributions 1,6].

Community involvement and access to responsive care may support building and maintaining meaningful connections, but migration barriers or geographic isolation can limit these opportunities [Contributions 4,5]. Ruan and Cheung [3] found that fewer connections outside family, poor-quality relationships and weaker community belonging were associated with loneliness among older adult migrants. Current evidence often considers these influences separately, leaving an incomplete understanding of how personal relationships and wider systems work together. The contributions to this Special Issue examine this gap across diverse populations and settings. This relational and contextual perspective provides a foundation for examining how each contribution advances current knowledge of social connection and well-being in later life.

## 3. Cross-Cutting Themes

The contributions from China and rural Peru show that social support is not a single uniform resource. In China, Cheng et al. [Contribution 6] found that psychological resilience partly explained the association between social support and self-reported well-being. These findings suggest that positive relationships may strengthen older adults’ ability to cope with stressors. Among older adults in rural Peru, Alvarado-García et al. [Contribution 1] further showed that varying forms of support may shape the relationship between stress and mental health in distinct ways. Affectionate support alongside positive social interactions partly explained the association between stress and depressive symptoms, while tangible assistance weakened that association. Together, these studies extend current understanding by showing that the influence of social connection depends on how support functions [1]. Social support may promote well-being by strengthening psychological resilience or providing resources that help older adults manage stress.

Not all forms of social contact were equally beneficial. Kokorelias et al. [Contribution 5] found that older adults living with HIV and a concurrent mental health diagnosis could still feel isolated despite actively socializing when they did not feel seen and understood. Meaningful relationships were based on authenticity and trust through shared experiences and judgment-free spaces, challenging the assumption that social activity alone protects against isolation. This distinction was also evident in older adults receiving care from migrant domestic workers in Hong Kong. Hung et al. [Contribution 2] found that the quality of relationships was associated with lower social loneliness and stronger perceived social networks. Yang et al. [Contribution 3] demonstrated that where the support came from played a key role. Across the early and later stages of the COVID-19 pandemic, support from friends rather than family predicted psychological well-being and life satisfaction among older adult immigrants. The different roles of friendship and family support show that the sources of support cannot be used interchangeably. Viewed through the Convoy Model, these findings show how the structure of a relationship network can shape the forms of support available and how they contribute to well-being [2]. Collectively, these findings emphasize the importance of relationships that provide emotional safety and shared understanding, rather than simply the number of social ties.

The contributions also highlight the community conditions that shape individual relationships. Lee [Contribution 7] found that community social capital was not associated with health equally across age groups. Sense of belonging was more strongly associated with health among adults aged 60 to 69, while neighbor communication and perceived regional inequality were more strongly associated with those aged 70 and older. These differences show that the relationship between community social capital and health varies throughout later life. Li et al. [Contribution 8] found that the presence of community organizations did not guarantee better mental health. Agricultural cooperatives and cultural and sports clubs were associated with better mental health. This association was not present through volunteer groups, senior dance troupes, or senior associations. The beneficial associations were found through organizations with greater involvement in daily life, alongside practical and economic value. These studies shift attention to the relevance of community resources, suggesting that well-being may be effectively supported when the forms of socialization adapt to older adults’ changing needs and circumstances.

Opportunities for connection were also not equally accessible for people. Most older adult immigrants surveyed by Guruge et al. [Contribution 4] wanted greater community participation but faced barriers such as language, transportation, and not wanting to attend alone. Reliance on family reflected barriers that prevented participants from expanding their social networks. This suggests that lower participation may not be a lack of interest in forming connections but reflects barriers to access. These findings add context to broader research on loneliness among older adult migrants by identifying practical barriers that may restrict connections beyond the family [3]. There was similar evidence of geographic inequalities in Kokorelias et al. [Contribution 5], where living in suburban and rural residences could potentially intensify the isolation among older adults living with HIV and a concurrent mental health diagnosis. Virtual spaces helped some participants overcome distance and mobility limitations, but limited digital literacy created new barriers. Yang et al. [Contribution 3] found that life satisfaction, psychological well-being and social support among Chinese older adult immigrants were lower during later stages of the COVID-19 pandemic than during its early stage. The lower levels found in the later stages show that disruptions to social connection may continue beyond periods of physical distancing. Social connections may be difficult to sustain with limited opportunities for participation and a lack of culturally relevant, digitally inclusive support.

Caregiving relationships emerged as an important, though often undervalued, source of social support. Hung et al. [Contribution 2] found that migrant domestic workers supported older adults by providing companionship, facilitating outings, and helping them remain connected to community life. These benefits depended on relationship quality, which could be constrained by employment hierarchies. Haghiri-Vijeh et al. [Contribution 9] also emphasized the value of recognition by explaining that migrant 2SLGBTQIA+ unpaid caregivers, particularly chosen-family members, may be excluded from care decisions even when providing consistent emotional and practical support. This exclusion shows how institutional definitions of family can weaken functioning sources of care. Al-Hamad et al. [Contribution 10] similarly found that family caregivers often lack adequate training or financial assistance, while also bridging cultural and healthcare access gaps for older adult migrants. Collectively, these studies show that caregiving relationships are shaped by wider systems, and not only by the support provided within them. Without adequate recognition and support, caregivers may need to maintain connection and care with additional strain.

These findings also indicate that responsive support should not be limited to individual interactions but should also be built into service design. Language assistance, transportation, and partnerships with community organizations were identified in Al-Hamad et al. [Contribution 10] and Guruge et al. [Contribution 4] as important for making social participation accessible. Yang et al. [Contribution 3] reinforced the value of sustaining community programming to help Chinese older adult immigrants develop friendships and rebuild social support following the COVID-19 pandemic. Haghiri-Vijeh et al. [Contribution 9] showed that provider education, support programs, and institutional policies must consider migrant and 2SLGBTQIA+ identities alongside more inclusive understandings of family. These findings show that maintaining social connection is more than an individual responsibility. Recent evidence suggests that interventions combining multiple approaches may be more effective than those focused on a single objective, although rigorous testing remains necessary [4]. To support lasting connections, services should make participation accessible and responsive to the circumstances of the community they serve.

## 4. Conclusions

The contributions to this Special Issue demonstrate that social connection in later life is not defined simply by contact frequency or network size. Its relationship with well-being depends on the quality and function of relationships, opportunities for meaningful participation, and broader community and service conditions [1]. Across diverse populations and settings, the studies identify emotional safety [Contribution 5], trust [Contributions 2,5], practical support [Contributions 1,9], belonging [Contribution 7], and accessible community resources as important dimensions of connection [Contributions 3,10]. They also show how migration, geography, language, inequality, and unrecognized caregiving relationships can restrict access to these resources. These contributions advance current understanding by showing that the relational and contextual aspects of social connection must be taken together when examining well-being in later life.

These findings call for responses that extend beyond addressing loneliness at the individual level. Healthcare providers, community organizations, and policymakers should work together to develop culturally responsive, geographically accessible, and relationship-centered supports that recognize diverse identities, families, and caregiving arrangements. Future research should examine how relationship quality, support, and access to community resources interact across different populations and settings. It should also evaluate whether accessible and relationship-centered supports can create and maintain meaningful connections [4]. In the post-pandemic context, rebuilding social connections should be understood as a shared responsibility across relationships, communities, and systems.

## Supplementary Materials

The following supporting information can be downloaded at: https://www.mdpi.com/article/10.3390/healthcare14183048/s1, Table S1: Overview of the contributions to the Special Issue.
