# Supplementary material for: Impact of Social Connections on Well-Being of Older Adults: Relationships, Communities, and Systems of Support
Source: Healthcare (Basel). 2026 Sep 17;14(18):3048. doi: 10.3390/healthcare14183048 (PMC13606726; doi:10.3390/healthcare14183048)
Supplement: Supplementary file 1 [file healthcare-14-03048-s001.zip › healthcare-4594296-supplementary.pdf]

Table S1. Overview of the contributions to the Special Issue

Contribution	Context and Sample	Objectives	Methods	Key findings and implications
Cheng et al., "Social Support and Well-Being Among Community-Dwelling Older Adults: The Mediating Role of Psychological Resilience"	1,227 adults aged 65 and older living in three urban communities in Dongguan, China.	Examine the relationship between social support and well-being and whether psychological resilience helped explain this relationship.	A cross-sectional survey measuring social support, psychological resilience, and well-being. Correlation and mediation analyses were conducted.	Greater social support and psychological resilience were associated with better well-being. The relationship between social support and well-being was partly explained by psychological resilience. Community and primary care programs could strengthen social connections and psychological resilience among older adults.
Lee, "Spatial Inequality, Community Social Capital, and Age-Differentiated Health Vulnerabilities Among the Elderly in South Korea: A Hierarchical Linear Modeling Approach"	1,020 adults aged 60 and older across 17 metropolitan cities and provinces in South Korea.	Investigate how community social capital and perceived regional inequality were associated with health across different older age groups.	Cross-sectional analysis of the 2024 Social Integration Survey. Hierarchical linear models and age-group comparisons were conducted.	A stronger sense of community belonging was associated with better self-rated health. Perceived regional inequality was associated with poorer health. Belonging was especially important among adults aged 60-69, while neighbour communication was important among those aged 70 and older. Community care programs should address the needs of each age group.
Li et al., "Rural Community Organizations and Mental Health Among Older Adults: Evidence of Dual Economic-Social Pathways in Rural China"	511 adults aged 60 and older living in rural Anhui Province, China.	Examine how different rural community organizations were associated with depressive symptoms and life satisfaction.	Longitudinal panel study using survey data collected in 2015, 2018, and 2021. Two-way fixed effects models were used to analyze the data.	Agricultural cooperatives were linked to fewer depressive symptoms. Cultural and sports clubs were linked to fewer depressive symptoms and greater life satisfaction. These associations reflected improved financial stability and stronger social connections. Supporting organizations that combine economic and social functions may benefit older adults' mental health.
Alvarado-García et	166 adults	Determine	A cross-	Greater stress was associated with

al., “Perceived Social Support and Well-Being: Mediation and Buffering of the Stress–Depression Link in Rural Older Adults”	aged 60 and older living in a rural coastal district in northern Peru.	whether perceived social support explained or weakened the relationship between stress and depressive symptoms.	sectional survey measuring stress, depressive symptoms, and four types of social support.	more depressive symptoms. Positive social interaction and affectionate support helped explain this relationship. Tangible support weakened the link between stress and depression. Meaningful social connections and practical assistance should be provided to support the mental health of older adults.
Kokorelias et al., “Lived Experiences of Social Isolation and Meaningful Relationships Among Older Adults Living with HIV with a Concurrent Mental Health Diagnosis: A Heideggerian Phenomenological Approach”	33 adults aged 50 and older in Ontario, Canada who were living with HIV and a diagnosed mental health condition.	Explore how older adults living with HIV and a mental health condition experience social isolation and develop meaningful relationships.	In-depth, semi-structured interviews. The data were analyzed using descriptive and interpretive coding guided by Social Convoy Theory.	Social isolation involved both physical separation and feeling unseen or misunderstood. Meaningful relationships were built on emotional safety and authenticity, especially among peers who share similar experiences. Virtual platforms supported this connection, while rural settings often increased isolation. Affirming, context-sensitive spaces that support trust and reciprocal relationships may help strengthen meaningful social connections.
Guruge et al., “Social Participation Among Older Immigrants: A Cross-Sectional Study in Nine Cities in Canada”	476 Arabic, Mandarin, and Punjabi-speaking immigrants aged 60 and older across nine Canadian cities.	Examine the social activities older immigrants participated in, their desire for greater participation, and the barriers they experienced.	A cross-sectional questionnaire. Descriptive statistics were used to examine social participation.	Most participants engaged in social activities involving family and friends, while community activities occurred infrequently. Most of them wanted greater social participation, but faced barriers such as language and not wanting to attend alone. Programs with language and transportation support may improve community participation among older immigrants.
Hung et al., “Loneliness and Social Isolation of Older Adults and Quality of Dyadic Relationships with Migrant Domestic	288 adults in Hong Kong aged 60 and older who lived with and	Examine how the quality of the relationships with migrant domestic workers was	cross-sectional survey followed by in-depth interviews and content	Better relationships with migrant domestic workers was associated with less social loneliness. Trust, companionship and support with social activities helped older adults remain connected. Training and support for migrant domestic

Workers: A Mixed-Method Study”	received care from migrant domestic workers.	associated with loneliness and explore older adults’ experiences of care.	analysis.	workers may strengthen relationship-centred care.
Yang et al., “Psychological Wellbeing and Life Satisfaction among Chinese Older Immigrants in Canada across the Early and Late Stages of the COVID-19 Pandemic”	Two independent samples of Mandarin-speaking Chinese immigrants in Canada aged 65 and older.	Compare psychological well-being, life satisfaction, and perceived social support during the early and late stages of the COVID-19 pandemic.	A cross-sectional survey, Group comparisons and hierarchical regression analyses were conducted.	Psychological well-being, life satisfaction, and perceived social support were lower in 2023 than in 2020. Support from friends predicted greater well-being and life satisfaction. The findings support sustained mental health services and community programs that promote friendship among older Chinese immigrants.
Haghiri-Vijeh et al., “Supporting Migrant 2SLGBTQIA+ Unpaid Caregivers for Family Members Living with Chronic Illnesses”	Migrant 2SLGBTQIA+ people providing unpaid care to family members or chosen family living with chronic illnesses.	Describe how intersecting migrant and 2SLGBTQIA+ identities shape unpaid caregiving experiences and support needs.	Narrative review of peer-reviewed research, policy reports, systematic reviews, and organizational fact sheets.	Migrant 2SLGBTQIA+ caregivers faced overlapping discrimination and multiple barriers contributing to emotional and physical strain. Caregivers within chosen families were often excluded from care decisions. Healthcare systems should improve provider education and collect inclusive data to develop more responsive services and policies.
Al-Hamad et al., “Home Healthcare Among Aging Migrants: A Joanna Briggs Institute Scoping Review”	Home healthcare experiences of migrants and refugees aged 65 and older. The review included 35 sources from nine countries.	Summarize current evidence on aging migrants’ experiences with home healthcare and its influence on their health and well-being.	Scoping review of sources published from 2000 to 2024. Findings were organized using content analysis.	Language barriers, cultural differences, and systemic barriers obstructed access to home healthcare. Families frequently helped to fill service gaps but experienced emotional and financial strain. Culturally responsive services helped build trust and preserve cultural identity. Provider training and formal support for family caregivers may improve access and care quality.

As shown in Table S1, the ten contributions included studies from multiple geographic settings and methodological approaches. Of the eight empirical studies, four were conducted in East Asia, including two in China, one in South Korea, and one in Hong Kong. Three were conducted in Canada, while one was conducted in rural Peru. Six studies used quantitative methods, one used qualitative methods, and one used mixed methods. The last two contributions included a narrative review and a scoping review.
